# PAH–DNA Adducts, Cigarette Smoking, *GST* Polymorphisms, and Breast Cancer Risk

**DOI:** 10.1289/ehp.0800119

**Published:** 2008-12-10

**Authors:** Kathleen M. McCarty, Regina M. Santella, Susan E. Steck, Rebecca J. Cleveland, Jiyoung Ahn, Christine B. Ambrosone, Kari North, Sharon K. Sagiv, Sybil M. Eng, Susan L. Teitelbaum, Alfred I. Neugut, Marilie D. Gammon

**Affiliations:** 1 Department of Epidemiology, University of North Carolina at Chapel Hill, Chapel Hill, North Carolina, USA;; 2 Department of Epidemiology and Public Health, Division of Environmental Health Sciences, School of Medicine, Yale University, New Haven, Connecticut, USA;; 3 Department of Environmental Health Sciences, Mailman School of Public Health, Columbia University, New York, New York, USA;; 4 Department of Epidemiology and Biostatistics, Arnold School of Public Health, University of South Carolina, Columbia, South Carolina, USA;; 5 Division of Cancer Epidemiology and Genetics, National Cancer Institute, Bethesda, Maryland, USA;; 6 Department of Cancer Prevention and Control, Roswell Park Cancer Institute, Buffalo, New York, USA;; 7 Epidemiologic Resources, Safety Evaluation, and Epidemiology, Pfizer, Inc., New York, New York, USA;; 8 Department of Community and Preventive Medicine, Mount Sinai School of Medicine, New York, New York, USA;; 9 Department of Epidemiology, Mailman School of Public Health and; 10 Department of Medicine, College of Physicians and Surgeons, Columbia University, New York, New York, USA

**Keywords:** breast cancer, GST, Long Island, PAH-DNA adducts, smoking

## Abstract

**Background:**

Polycyclic aromatic hydrocarbons (PAHs) may increase breast cancer risk, and the association may be modified by inherited differences in deactivation of PAH intermediates by glutathione *S*-transferases (GSTs). Few breast cancer studies have investigated the joint effects of multiple GSTs and a PAH biomarker.

**Objective:**

We estimated the breast cancer risk associated with multiple polymorphisms in the *GST* gene (*GSTA1*, *GSTM1*, *GSTP1*, and *GSTT1*) and the interaction with PAH–DNA adducts and cigarette smoking.

**Methods:**

We conducted unconditional logistic regression using data from a population-based sample of women (cases/controls, respectively): *GST* polymorphisms were genotyped using polymerase chain reaction and matrix-assisted laser desorption/ionization time-of-flight assays (*n* = 926 of 916), PAH–DNA adduct blood levels were measured by competitive enzyme-linked immunosorbent assay (*n* = 873 of 941), and smoking status was assessed by in-person questionnaires (*n* = 943 of 973).

**Results:**

Odds ratios for joint effects on breast cancer risk among women with at least three variant alleles were 1.56 [95% confidence interval (CI), 1.13–2.16] for detectable PAH–DNA adducts and 0.93 (95% CI, 0.56–1.56) for no detectable adducts; corresponding odds ratios for three or more variants were 1.18 (95% CI, 0.82–1.69) for ever smokers and 1.44 (95% CI, 0.97–2.14) for never smokers. Neither interaction was statistically significant (*p* = 0.43 and 0.62, respectively).

**Conclusion:**

We found little statistical evidence that PAHs interacted with *GSTT1*, *GSTM1*, *GSTP1*, and *GSTA1* polymorphisms to further increase breast cancer risk.

Polycyclic aromatic hydrocarbons (PAHs) are an environmentally ubiquitous class of compounds that are formed during incomplete combustion of organic substances. Several PAHs have been classified as probable human carcinogens (Boffetta et al. 1997; Shimada 2006). In the general population, the major source of exposure is tobacco smoke (Lioy and Greenberg 1990), with diet (grilled/smoked foods, cereals, and leafy green vegetables) and exposure to fossil fuel combustion by-products contributing to cumulative exposure (Waldman et al. 1991). Although there is strong evidence for the relationship between PAH exposure and lung, skin, and bladder cancer in humans, it is equivocal as to whether PAH exposure is associated with breast cancer (Boffetta et al. 1997; Gammon and Santella 2008; Shimada 2006).

PAH–DNA adducts reflect not only PAH exposure but also the body’s ability to metabolize these compounds. The formation of these carcinogen–DNA adducts is recognized as a key event in the initiation of carcinogenesis (Gammon et al. 2004b; Hecht 2003; Wogan et al. 2004). In the Long Island Breast Cancer Study Project (LIBCSP), a large population-based case–control study, blood levels of PAH–DNA adducts were associated with a modest 29–35% elevation in breast cancer risk among women (Gammon et al 2004b). These findings are consistent with previously conducted, smaller hospital-based studies (Li et al. 1996, 2002; Rundle et al. 2000a). Animal studies have provided further support for the role of PAHs as mammary carcinogens (Cavalieri et al. 1991; el-Bayoumy et al. 1995). Although a significant association between breast cancer risk and detectable PAH–DNA adducts has been noted, a dose–response relationship was not observed in the LIBCSP. Further stratification by known sources of PAH exposure, such as cigarette smoking or the consumption of PAH-containing foods, did not modify this association (Gammon et al. 2004b). These findings are consistent with those from smaller studies (Hu et al. 2007). The lack of a dose response effect suggests that there may be subgroups of women who are more genetically susceptible to the carcinogenic effects of PAH exposure on breast tissue.

Inherited differences in the metabolism of PAHs, interindividual differences in enzyme expression, and induction may be key determinants in individual breast cancer susceptibility (Shimada 2006). Genetic differences in metabolic activation and detoxification of PAHs have been shown to affect differences in phenotypic biomarkers such as urinary metabolites and are hypothesized to influence cancer risk (Brescia et al. 2004; Hatsukami et al. 2006; Hecht et al. 2006; Kuljukka-Rabb et al. 2002; Upadhyaya et al. 2006; Wogan et al. 2004). Further investigation of these genetic differences in the deactivation of PAH metabolites may contribute to our understanding of the lack of a dose–response relationship and increased understanding of breast cancer risk.

The phase II metabolic super family of GST enzymes is involved in the metabolic activation and deactivation of PAH metabolites, and polymorphisms in these genes lead to specific changes in enzyme function and the capacity to metabolize PAH compounds (Hecht 1999). The *GSTM1* null polymorphism [GenBank BC036805; National Center for Biotechnology Information (NCBI) 2009a] results in the absence of isoenzyme (mu) expression and reduced glutathione binding efficiency of PAH epoxides and other genotoxic substrates (Hayes and Pulford 1995). The *GSTP* 1le105Val polymorphism [*GSTP1*; Unigene Hs.523836 (NCBI 2009b)] is associated with reduced isoenzyme (pi) expression and a reduced capacity to inactivate carcinogens, including the diol epoxides created during phase I metabolism of PAHs (Coles and Kadlubar 2003; Pavanello and Clonfero 2000; Ryberg et al. 1997; Watson et al. 1998). The *GSTT1* null polymorphism (Genbank X79389) results in the absence of (theta) isoenzyme (Hayes and Pulford 1995), but the role of this polymorphism in relation to PAH metabolism is uncertain (Pavanello and Clonfero 2000). On the one hand, the theta isoenzyme is thought to activate alkylating agents, such as nitrosamines present in tobacco smoke (Hecht 1999; Pavanello and Clonfero 2000); on the other hand, in human erythrocytes, the theta isoenzyme binds reactive conjugates to glutathione, inhibiting genotoxic damage to DNA (Pelin et al. 1996). GSTs conjugate PAH diol epoxides into water-soluble products, and GST enzymes also protect against products of oxidative stress (Mitrunen and Hirvonen 2003). *GSTA1* (Unigene Hs. 446309) is thought to decrease reactive oxygen species and, via an increase in antioxidant levels, to also play an active role in PAH metabolism (Ahn et al. 2006; Coles et al. 2005; Korashy and El-Kadi 2006). For *GSTA1*, two alleles, hGSTA1*A and hGSTA1*B, differ in three linked base changes in the 5′ promoter region of the gene: T→G at nucleotide 567, C→T at nucleotide 69, and G→A at nucleotide 52 (Coles et al 2001a, 2001b). These substitutions result in differential expression (Coles et al. 2001b), with lower transcriptional activation with GSTA1*B (variant) than with GSTA1*A (common) alleles. It has been demonstrated by our group that the variant GSTA1*B/*B* genotype is associated with higher risk of breast cancer among smokers compared with nonsmokers with the *A*/A* genotype (Ahn et al. 2006).

Only one breast cancer study (Rundle et al. 2000b) has reported on a potential interaction between *GSTM1* null genotype and PAH–DNA adducts. However, the sample size was small (< 100 cases), yielding unstable effect estimates and prohibiting exploration of more than one single nucleotide polymorphism from among the many that contribute to this complex cascade of activation and detoxification. Investigating whether genetically determined differences in multiple *GST* genotypes modify the association between breast cancer risk and PAH exposure may improve our ability to identify women who are susceptible to the carcinogenic effects of PAHs on breast tissue.

The goal of our present analysis was to investigate whether multiple *GST* polymorphisms modified the relationship between PAH exposure and breast cancer, using the lymphocyte PAH–DNA adduct biomarker as our primary exposure measure of interest using a large, population-based sample of cases and controls from the LIBCSP. We also evaluated the interaction between *GST* polymorphisms and smoking status, because smoking behavior was previously identified as the strongest predictor of detectable PAH–DNA adducts (Shantakumar et al. 2005). Finally, we investigated the joint effects of smoking status and *GST* polymorphisms on detectable PAH–DNA adducts among control women.

## Methods

### Study population

The population-based sample of cases and controls from the LIBCSP has been described previously (Gammon et al. 2002a). Briefly, cases were defined as English-speaking adult women newly diagnosed with breast cancer residing in Nassau County or Suffolk County in Long Island, New York. Population-based controls were identified through random-digit dialing for women < 65 years of age, and by Health Care Finance rosters for women ≥ 65 years of age within the same counties, and frequency matched them (by 5-year age groups) to cases. Participants were between 20 and 98 years of age; 94% were white, 4% were black, and 2% other races/ethnicities. The study protocol was approved by the institutional review boards of the collaborating institutions, and written informed consent was obtained from the LIBCSP participants.

### Data collection

Respondents to the case–control interview included 1,508 cases and 1,556 controls (Gammon et al. 2002a). The 2-hr in-person structured interview collected data on history of cigarette smoking (current, past), exposure to environmental tobacco smoke in the residential home throughout the life course (Gammon et al. 2004a), and other characteristics potentially relevant to breast cancer risk. Blood samples were obtained from 73% of cases and 73% of controls (Gammon et al. 2004b).

### Laboratory assays

We processed the blood samples collected by Gammon et al. (2004b), isolated the DNA and completed genotyping for *GSTT1* and *GSTM1* as previously described (Steck et al. 2007b), by a multiplex polymerase chain reaction method as previously described (Bell et al. 1992). Genotyping for *GSTP1* (Ile105Val; rs1695) (Steck et al. 2007b) and *GSTA1* (Ahn et al. 2006) was completed as previously described by matrix-assisted laser desorption/ionization time-of-flight assay (Fannon 2002). We included positive and negative controls in each batch, and a random 10% repeated sampling yielded a 97% concordance. Genotype distributions of *GSTA1* (*p* = 0.81) and *GSTP1* (*p* = 0.38) were in Hardy-Weinberg equilibrium among controls. The number of cases and controls, respectively, for whom genotyping was successful varied with genotype (*n* = 1,041 and 1,090 for *GSTA1*, 983 and 1,016 for *GSTT1*, 975 and 1,001 for *GSTM1*, and 1,027 and 1,069 for *GSTP1*). Missing genotype data were primarily due to insufficient DNA (Ahn et al. 2006; Steck et al. 2007b).

PAH–DNA adducts were measured in peripheral mononuclear cell DNA using competitive enzyme-linked immunosorbent assay as previously described (Gammon et al. 2002b). PAH–DNA adduct measurements were available for 873 cases and 941 controls who donated sufficient blood volume for the assay, as described previously (Gammon et al. 2004b).

### Statistical methods

We calculated odds ratios (ORs) and 95% confidence intervals (CIs) using unconditional logistic regression using SAS version 9.0 (SAS Institute Inc., Cary, NC). All models were adjusted for age at reference (date of diagnosis for cases and date of identification for controls), the frequency-matching factor. Covariates considered as potential confounders included race, family history of breast cancer, parity, age at menarche, age at first pregnancy, menopausal status, lifetime alcohol intake, education, and smoking status. We considered a covariate a confounder and included it in the models if it changed the ORs by more than 10% (Hosmer and Lemeshow 1989); however, adjustment by these potential confounders did not appreciably change the ORs, and therefore only the age-adjusted estimates are shown.

For our first set of analyses, our goal was to determine whether breast cancer risk is associated with the *GST* polymorphisms and whether they interact with PAH exposures, either alone or jointly, to affect breast cancer risk. We previously reported that the ORs for the association between breast cancer and individual *GST* genotypes were not substantially elevated (Ahn et al. 2006; Steck et al. 2007b), although risk was elevated when we considered multiple *GST*s (Ahn et al. 2006; Steck et al 2007b). Here, we focus primarily on the analyses that examine the interaction of *GST*s with adducts and smoking, using the following methods.

We estimated the ORs for breast cancer in relation to potential interactions between various PAH measures and the *GST* polymorphisms, *a*) considering each polymorphism separately (Table 1), and (*b*) by grouping individuals based on the number of variant alleles for any of the four *GST* polymorphisms considered (Tables 2 and 3). For this group of analyses, the reference group included women with *GSTT1* present, *GSTM1* present, *GSTP1* common (*AG* or *GG*), or *GSTA1* common (*A*/A*) genotypes. Using this approach, we explored the effect of even a single at-risk genotype, among those who carried common alleles for the other *GST*s considered. We chose to group the genes this way based on experimental evidence indicating reduced or no enzyme activity for the variant and null genotypes. In terms of metabolism, the *GSTP1* valine genotype is thought to be associated with lower glutathione *S*-transferase pi enzyme activity compared with the *GSTP1* isoleucine genotype, which plays a key role in the detoxification of benzo[*a*]pyrene diol epoxide, a major carcinogen present in tobacco smoke (Fields et al. 1998; Hecht 1999; Nakajima et al. 1995). To explore these potential gene–PAH interactions, we developed separate models for women based on the presence or absence of the PAH exposure measure [PAH–DNA adducts (detectable/not detectable); self-reported cigarette smoking status (ever/never, or current/past/never)] or pack years of smoking history (< 15 years, 15–30 years, > 30 years). We also developed models for all women that included multiplicative interaction terms (Hosmer and Lemeshow 1989); gene–environment interactions were formally assessed by examining departures from multiplicity by comparing models with and without the interaction term using the likelihood ratio test (LRT) (Hosmer and Lemeshow 1989). In addition, we investigated potential interactions on the additive scale (Hosmer and Lemeshow 1989) using joint indicator terms for genotype only, for PAH measure only, and for both genotype and PAH measure (Tables 3 and 4). Interaction contrast ratios (ICRs) and their 95% CIs were calculated to measure the relative excess risk due to interaction (Assmann et al. 1996; Rothman and Greenland 1998). We also considered other categorizations of smoking exposure status that incorporated information collected by the questionnaire on environmental tobacco smoke (Gammon et al. 2004a), but the cell sizes were too small to yield meaningful results, so we do not show these data.

For our second set of analyses, our goal was to determine the association between PAH–DNA adducts and the *GST* variant alleles among control women only. We used unconditional logistic regression with detectable adducts as the dependent variable, and *GST*s as the independent variable, controlling for age at reference and season of blood draw (Table 4). We also investigated the main effect of each genotype or number of variant alleles on the outcome of detectable PAH–DNA adducts in a multivariate model adjusted for age, smoking status, and season of blood draw. In a previous study (Shantakumar et al. 2005), we found that cigarette smoking was a significant predictor of detectable PAH–DNA adducts among this population-based sample; thus, we also explored whether the *GST* genotypes modified the association between smoking status and detectable adducts. We also considered potential interactions with cigarette smoke because of its link to our biomarker, although we recognize that other PAH sources and other constituents of tobacco smoke could potentially interact with *GST* polymorphisms to affect risk; however, these other constituents of tobacco smoke have not been linked to breast cancer risk in a human study.

## Results

### Individual GSTs, PAHs, and breast cancer risk

As shown in Tables 1 and 2, we did not observe substantial effect modification, on a multiplicative or additive scale, by any of the individual genotypes on the associations between breast cancer risk and PAH exposure (PAH–DNA adducts or cigarette smoking).

### Multiple GSTs and breast cancer risk

The ORs for the association between breast cancer risk and multiple *GST* variant alleles were 1.10 (95% CI, 0.77–1.57) for three or more variants, 0.76 (95% CI, 0.56–1.03) for two variants, and 0.80 (95% CI, 0.59–1.08) for one variant. When we considered the combined effects of *GSTM1*, *-P1*, *-T1*, and -*A1* variants that are coded based on their PAH-related biologic activity (rather than for their isothiocyanate-related activity), we did not find a significant association between the number of variant *GST* alleles and breast cancer risk.

### Multiple GSTs, PAHs, and breast cancer risk

Table 3 illustrates the potential joint effects of PAH–DNA adducts and the number of *GST* polymorphisms (one, two, or three or more variants) and breast cancer risk. We observed a 56% increase in risk (OR = 1.56; 95% CI, 1.13–2.16) among women with detectable adducts and three or more *GST* variants [*GSTT1* null variant, *GSTM1* null variant, *GSTP1 AG*/*GG* genotype, *GSTA1* common (A*/B* or B*/B*) genotypes] compared with those with all common genotypes and no detectable PAH–DNA adducts. The corresponding OR for three or more variants and no detectable adducts was 0.93 (95% CI, 0.56–1.56). Women with one variant allele and no detectable adducts had a 44% reduced risk of breast cancer compared with women with all common alleles and no detectable adducts (OR, 0.56; 95% CI, 0.40–0.77); the corresponding OR for detectable adducts was not significant (OR, 0.97; 95% CI, 0.78–1.20; Table 3).

As shown in Table 4, the ORs for the association between breast cancer risk and the number of *GST* variant alleles varied little with smoking status, and results for interaction on either a multiplicative or additive scale were not statistically significant. For example, the OR associated with three or more *GST* variants was 1.18 among ever smokers (95% CI, 0.82–1.69) but was 1.44 among never smokers (95% CI, 0.97–2.14). Results for the interaction between multiple *GST* variants and pack-years of smoking (data not shown) are similar to those shown in Table 4.

When we categorized ever smokers by whether they were current or past smokers, the OR for the association between breast cancer risk and three or more *GST* variants was 0.62 (95% CI, 0.27–1.43) among current smokers, and 1.66 (95% CI, 0.93–2.97) among past smokers, compared with never smokers with all four common alleles. However, the category sizes upon which these estimates are based were small (data not shown), and the *p*-value for the interaction on a multiplicative scale was 0.31.

### GSTs, smoking, and PAH–DNA adducts among control women

We did not observe a significant association between any single *GSTT1*, *GSTM1*, *GSTP1*, or *GSTA1* polymorphism and detectable PAH–DNA adducts among the control women (*GSTT1*: OR, 1.09; 95% CI, 0.77–1.56; *GSTM1*: OR, 0.93; 95% CI, 0.69–1.25; *GSTP1*: OR, 0.95; 95% CI, 0.72–1.27; *GSTA1*: OR, 1.15; 95% CI, 0.86–1.53) or the number of variant genotypes and detectable PAH–DNA adducts (one variant compared with all common alleles: OR, 1.60; 95% CI, 0.92–2.79; two variants compared with all common alleles: OR, 1.35; 95% CI, 0.77–2.36; three or more variants compared with all common alleles: OR, 1.45; 95% CI, 0.75–2.82). Table 5 shows the association between *GST* polymorphisms and PAH–DNA adducts among control women stratified by smoking status. Women who ever smoked and had the *GSTM1* present genotype had a 35% reduced odds (OR, 0.65; 95% CI, 0.45–0.93) of having detectable PAH–DNA adducts compared with never smokers with the same genotype. We noted similar results forever smokers with the *GSTM1* null genotype compared with never smokers with the *GSTM1* common genotype (OR, 0.64; 95% CI, 0.43–0.93). Ever smokers with the *GSTT1* present genotype had a 27% reduced odds of PAH–DNA adducts (OR, 0.73; 95% CI, 0.53–0.99) compared with never smokers with the same genotype. Ever smokers with the common *GSTA1* genotype had a reduced odds of PAH–DNA adducts compared with never smokers with the same genotype (OR, 0.67; 95% CI, 0.47–0.96; Table 5). We found no evidence of effect modification between any of the individual genotypes and smoking status on PAH–DNA adduct status on the multiplicative scale (LRT *p*-values: *GSTT1*, 0.20; *GSTM1*, 0.75; *GSTP1*, 0.15; *GSTA1*, 0.40) or on the additive scale. We did not find statistical evidence between number of variant alleles and smoking status on the odds of developing detectable PAH–DNA adducts on the additive scale (one-variant allele: ICR, 0.28; 95% CI, –0.39 to 0.45; two-variant alleles: ICR, –0.091; 95% CI, –0.57 to 0.38; three or more variant alleles: ICR, 0.177; 95% CI, –0.23 to 0.59) or on the multiplicative scale (*p*-value from the LRT = 0.13). When considering the number of adducts as a continuous outcome (data not shown), we found no evidence of a main effect of the individual genotypes on the number of adducts in an age-adjusted model (*GSTT1 p* = 0.86, *GSTM1 p* = 0.54, *GSTP1 p* = 0.91, *GSTA1 p* = 0.86).

## Discussion

To the best of our knowledge, this is the first epidemiologic investigation of breast cancer to consider the joint effects of four *GST* polymorphisms and several measures of PAH exposure, including a PAH–DNA adduct biomarker. Despite the biologic plausibility of our hypothesis, we found no strong evidence for an interaction between the number of variant *GST* alleles with various measures of PAH exposure (including ever/never smoking status, pack-years of smoking, or detected PAH–DNA adducts) and breast cancer risk, nor did we find an association among the control women between number of variant *GST* polymorphisms and the outcome of detectable PAH–DNA adducts. We found some evidence for a reduced risk of detectable adducts among control women who were ever smokers regardless of whether they had the *GSTM1* deletion polymorphism or the present genotype (Table 5). We also report for the first time that among controls with the A*/A* GSTA1 genotype, ever smokers had lower odds of having detectable PAH–DNA adducts than did never smokers with the same genotype (OR, 0.62; 95% CI, 0.41–0.92).

### GST polymorphisms and breast cancer risk

A few previous studies (Helzlsouer et al. 1998; van der Hel et al. 2005) including our own have found that individual polymorphisms in *GSTA1* (Ahn et al. 2006), *GSTM1* null (Helzlsouer et al. 1998; Spurdle et al. 2007), and *GSTT1* null (van der Hel et al. 2005) and *GSTP1* (heterozygote or homozygote valine) (Helzlsouer et al. 1998) are modestly associated with breast cancer, although others have reported no association with *GSTT1* (García-Closas et al. 1999; Helzlsouer et al, 1998; Vogl et al. 2004), *GSTP1* (Vogl et al. 2004), or *GSTM1* (Ambrosone et al. 1995; García-Closas et al. 1999; Vogl et al. 2004). Effects of multiple combinations of *GST* polymorphisms on breast cancer risk have previously been conducted in smaller studies, with conflicting findings (Helzlsouer et al. 1998; Steck et al. 2007b; van der Hel et al. 2005; Vogl et al. 2004). Combined effect of all three *GSTT1*, *GSTM1*, and *GSTP1* variants have been reported to have greater than a 3-fold increase in breast cancer risk compared with women with the common genotype for all three polymorphisms (Helzlsouer et al. 1998; Steck et al. 2007b). In this analysis, in which we considered the combined effects of the *GSTT1*, *-M1*, *-P1*, and -*A1* variants that are coded based on their PAH-related biologic activity (rather than for their isothiocyanate-related activity), we did not find a significant association between the number of variant *GST* alleles and breast cancer risk.

### GST polymorphisms, PAHs, and breast cancer

Previous studies have differed on whether *GST* polymorphisms modify the association between cigarette smoking and breast cancer risk (Millikan et al. 2000; Terry and Goodman 2006; van der Hel et al. 2005; Vogl et al. 2004; Zheng et al. 2002). Smaller studies have reported that smoking did not modify associations between *GSTT1* (Vogl et al. 2004), *GSTM1* (van der Hel et al. 2005; Vogl et al. 2004), or *GSTP1* polymorphisms (Vogl et al. 2004) and breast cancer, whereas former smokers with *GSTT1* null were found to have an increased risk of breast cancer compared with never smokers with *GSTT1* present (van der Hel et al. 2005). For example, one recent breast cancer meta-analysis reported no interaction between individual *GST* polymorphisms and smoking, whereas another meta-analysis reported positive associations between breast cancer risk and *GSTT1* present and *GSTM1* null genotypes among smokers (Terry and Goodman 2006; Vogl et al. 2004). In contrast, we observed an increased risk of breast cancer with three or more variant *GST* alleles in a previous analysis (Steck et al. 2007b), but no increased risk with number of variant alleles as defined by low PAH-metabolizing activity, and no evidence of gene–environment interaction with PAH exposure. Our present findings are unique in that we investigated four *GST* polymorphisms simultaneously in a single large study.

To the best of our knowledge, this is the first study to examine the interaction between PAH–DNA adducts and individual/combined effects of *GSTA1*, *GSTM1*, *GSTP1*, and *GSTT1* genotypes on breast cancer risk. We found no pronounced adverse effect for the genotype–PAH exposure interaction, regardless of the PAH measure. Given that the genetic polymorphisms we examined are known to be functionally associated with deactivation of the active intermediates of PAHs, our findings are not easily interpreted. It is possible that the PAH–DNA adduct biomarker effect estimates already reflect any effects associated with metabolic variation; however, if this were true, then any apparent interaction between PAHs and *GST*s should have been evident when examining the joint effects with smoking. We found a reduction in risk among women with at least one variant allele and no detectable adducts, but we did not find a similar reduction among these same women in relation to no smoking. Thus, we do not report evidence that PAHs interacted with these polymorphisms to further increase risk.

We acknowledge that expression of GSTs can be modulated by a variety of factors, including diet. There is extensive literature on animal studies demonstrating an interaction between isothiocyanates and PAHs on cancer risk, and the induction of phase II enzymes by isothiocyanates (Hecht 1996, 2000). The interaction between GSTs and isothiocyanates does not appear to interact to affect breast cancer risk in humans or the variations in the urinary metabolites of isothiocyanates in our data (Steck et al. 2007a, 2007b). Whether cruciferous vegetable intake would further interact with PAHs and GSTs to affect breast cancer risk is unclear. Unfortunately, this study was underpowered to explore such three way interactions despite a large sample size. Nevertheless, it may be theoretically possible that breast cancer risk is influenced by a potential three-way interaction of PAH–DNA adducts, *GST* polymorphisms, and cruciferous vegetable intake, although our current available data do not support this hypothesis (Steck et al. 2007a, 2007b).

### GST polymorphisms, smoking, and PAH–DNA adducts among control women

We considered the PAH–DNA adduct outcome as both a continuous and a dichotomous variable (detected/not detected), although the latter may be more informative given the lack of a dose–response relationship between adducts and breast cancer risk (Gammon et al. 2002b). In our analyses among control women, individual *GSTT1*, *GSTM1*, *GSTP1*, and *GSTA1* genotypes were not associated with detectable PAH–DNA adducts, nor was the number of variant *GST* alleles. Even after stratification on smoking, the number of variant *GST* alleles was not associated with detectable PAH–DNA adducts; however, ever smokers with either the *GSTM1* null or *GSTM1* present genotype had a reduced risk of detectable PAH–DNA adducts (Table 5). We observed a similar finding within the strata of ever smokers and the *GSTP1* common genotype compared with never smokers with the common genotype. Our results are the first published findings regarding *GSTA1* polymorphisms and PAH–DNA adducts. Our findings related to polymorphisms in *GSTM1* and PAH–DNA adduct formation are in contrast with one study (Rundle et al. 2003), while being consistent with others that report no significant difference in detectable PAH–DNA adducts based on *GSTM1* status (Grinberg-Funes et al. 1994; Rothman et al. 1995; Weiserbs et al. 2003). However, the LIBCSP results reported here are based upon a much larger sample size than previous analyses, which yields more stable effect estimates. Additionally, we used different methods to measure the PAH–DNA adducts in the various studies, possibly influencing the results.

### Methodological considerations

In an epidemiologic study, the use of a biomarker of exposure, such as PAH–DNA adducts, is considered superior because exposure assessment is not based on self-report, eliminating concerns for recall bias. Reliability in the measurement of genotype and adducts was high. Measurement error was possible, although unlikely with genotyping and analysis of adducts; however, because the laboratory was blinded to status of samples, any measurement error in measuring genotype and of PAH–DNA adduct status was independent of case–control status. Therefore, any resulting bias would have been nondifferential.

Our study power was limited when we examined interactions within subgroups of women, such as those with PAH–DNA adducts. However, the LIBCSP is the largest study conducted to date with information on PAH–DNA adducts with which to evaluate these associations, and thus our study power was better than that of previously reported studies.

We selected the *GST* polymorphisms because of their role in phase II metabolism, specifically metabolism of PAH-reactive intermediates. Because PAH–DNA adducts may be related to inherited differences in the metabolism of PAHs, this pathway was hypothesized to be important in individual breast cancer susceptibility. *GSTP1* and *GSTM1* have been shown to have a role in detoxification of PAH carcinogenic intermediates produced by cytochrome P450 in phase I metabolism (Butkiewicz et al. 2000). *GSTT1* and *GSTA1* have also been implicated in the metabolism of PAH intermediates (Garte et al. 2007; Hayes and Pulford, 1995; Korashy and El-Kadi 2006).

## Conclusion

Our previous analyses have shown that breast cancer risk was modestly elevated in relation to PAH–DNA adducts (Gammon et al. 2002b, 2004b), among certain subgroups of smokers (Gammon et al. 2004a), and among women with multiple variant alleles in *GST* genes (Steck et al. 2007b). In this analysis, however, we did not find strong evidence for further elevation of breast cancer risk, either on a multiplicative or on an additive scale, when we considered the joint effect of multiple GSTs and PAHs (cigarette smoking or PAH–DNA adducts), despite the biologic plausibility of such an interaction. These findings are based upon the largest population-based study of breast cancer conducted to date with a biomarker of PAH exposure. Further study may be warranted incorporating other biologic measures of PAH metabolism.

## Figures and Tables

**Table 1:** Age-adjusted ORs (95% CIs) for the individual *GST* genotypes on the associations between smoking status and breast cancer risk.

Genotype	Smoking status	Cases (*n* )	Controls (*n* )	OR (95% CI)	*p*-Value^a^
*GSTT1*					
Present	Never	351	341	1.0	0.27
	Ever	423	454	0.94 (0.80–1.11)	
Null	Never	97	112	0.97 (0.66–1.16)	
	Ever	112	109	1.04 (0.78–1.37)	

*GSTM1*					
Present	Never	227	247	1.0	0.62
	Ever	278	300	0.98 (0.81–1.19)	
Null	Never	218	201	1.14 (0.92–1.42)	
	Ever	252	253	1.06 (0.86–1.29)	

*GSTP1*					
*AA* (common)	Never	235	242	1.0	0.86
	Ever	275	309	0.91 (0.75–1.10)	
*AG* or *GG*	Never	239	234	1.04 (0.85–1.28)	
	Ever	278	284	1.01 (0.83–1.22)	

*GSTA1*					
A*/A* (common)	Never	145	168	1.0	0.60
	Ever	199	218	0.96 (0.77–1.20)	
A*/B* or B*/B*	Never	335	328	1.06 (0.88–1.28)	
	Ever	362	376	1.02 (0.85–1.22)	

Common alleles: *GSTT1* present, *GSTM1* present, *GSTP1* (*AA*), *GSTA1* (A*/A*). Variant alleles: *GSTT1* null, *GSTM1* null, *GSTP1* (*AG* or *GG*), *GSTA1* (A*/B* or B*/B*).

a From likelihood ratio test.

**Table 2:** Age-adjusted ORs (95% CIs) for the individual *GST* genotypes on the associations between PAH–DNA adducts and breast cancer risk.

Genotype	PAH–DNA adducts	Cases (*n*)	Controls (*n*)	OR (95% CI)	*p*-Value^a^
*GSTT1*					
Present	−	165	204	1.0	0.22
	+	463	474	1.01 (0.87–1.19)	
Null	−	40	61	0.68 (0.45–1.03)	
	+	134	124	1.14 (0.87–1.48)	

*GSTM*1					
Present	−	104	146	1.0	0.75
	+	301	320	0.99 (0.82–1.19)	
Null	−	100	114	0.93 (0.70–1.23)	
	+	293	274	1.13 (0.93–1.37)	

*GSTP1*					
*AA* (common)	−	107	133	1.0	0.77
	+	320	293	0.94 (0.78–1.13)	
*AG* or *GG*	−	106	153	0.83 (0.63–1.09)	
	+	304	333	1.15 (0.95–1.38)	

*GSTA1*					
A*/A*	−	75	111	1.0	0.53
	+	211	223	0.97 (0.78–1.20)	
A*/B* or B*/B*	−	145	179	0.83 (0.65–1.05)	
	+	416	413	1.03 (0.86–1.22)	

Common alleles: *GSTT1* present, *GSTM1* present, *GSTP1* (*AA*), *GSTA1* (A*/A*). Variant alleles: *GSTT1* null, *GSTM1* null, *GSTP1* (*AG* or *GG*), *GSTA1* (A*/B* or B*/B*). Symbols: +, PAH–DNA adducts detected; –, PAH–DNA adducts not detected.

a From likelihood ratio test.

**Table 3:** Age-adjusted ORs (95% CIs) for the effects of number of variant genotypes, PAH–DNA adducts, and risk of breast cancer.

No. of variants	PAH–DNA adduct status	Cases (*n*)	Controls (*n*)	OR (95% CI)	*p*-Value^a^	ICR (95% CI)
Four common	−	28	20	1.00		
	+	72	68	1.01 (0.71 to 1.43)		Referent
One variant	−	64	112	0.56 (0.40 to 0.77)	0.05	
	+	215	219	0.97 (0.78 to 1.20)		0.60 (0.23 to 0.97)
Two variants	−	74	93	0.78 (0.56 to 1.07)	0.18	
	+	183	222	0.82 (0.66 to 1.03)		0.36 (−0.12 to 0.84)
Three variants	−	29	31	0.93 (0.56 to 1.56)	0.43	
	+	105	66	1.56 (1.13 to 2.16)		0.75 (0.20 to 1.30)

Common alleles: *GSTT1* present, *GSTM1* present, *GSTP1* (*AA*), *GSTA1* (A*/A*). Variant alleles: *GSTT1* null, *GSTM1* null, *GSTP1* (*AG* or *GG*), *GSTA1* (A*/B* or B*/B*). Symbols: +, PAH–DNA adducts detected; –, PAH–DNA adducts not detected.

a From likelihood ratio test.

**Table 4:** Age-adjusted ORs (95% CIs) for the effect of number of variant genotypes, smoking status, and risk of breast cancer.

No. of variants	Smoking status	Cases (*n*)	Controls (*n*)	Total	OR (95% CI)	*p*-Value^a^	ICR (95% CI)
Four common	Never	58	39	97	1.0		
	Ever	65	66	131	0.98 (0.67 to 1.43)	0.24	Referent
One variant	Never	162	177	339	0.96 (0.73 to 1.25)		
	Ever	188	204	392	0.91 (0.71 to 1.18)	0.66	0.30 (−0.13 to 0.72)
Two variants	Never	139	164	303	0.89 (0.68 to 1.18)		
	Ever	179	204	383	0.88 (0.68 to 1.14)	0.52	0.32 (−0.10 to 0.73)
Three or more variants	Never	70	51	121	1.44 (0.97 to 2.14)		
	Ever	82	68	150	1.18 (0.82 to 1.69)	0.62	0.15 (−0.47 to 0.77)

Common alleles: *GSTT1* present, *GSTM1* present, *GSTP1* (*AA*), *GSTA1* (A*/A*). Variant alleles: *GSTT1* null, *GSTM1* null, *GSTP1* (*AG* or *GG*), *GSTA1* (A*/B* or B*/B*).

a From likelihood ratio test.

**Table 5:** Age-adjusted ORs (95% CIs) for the joint effect of *GST* polymorphisms and smoking on the outcome of detectable PAH–DNA adducts among control women.

	Smoking status	Adducts not detected	Adducts present	OR (95% CI)	*p*-Value^a^	ICR (95% CI)
Genotype						
*GSTT1*						
Present	Never	99	197	1.0	0.20	(−0.37 ((−1.04 to 0.31)
	Ever	105	277	0.73 (0.53 to 0.99)		
Null	Never	37	60	1.18 (0.74 to 1.87)		
	Ever	24	64	0.72 (0.43 to 1.20)		
*GSTM1*						
Present	Never	78	140	1.0	0.76	0.10 (−0.26 to 0.46)
	Ever	68	180	0.65 (0.45 to 0.93)		
Null	Never	56	115	0.85 (0.57 to 1.26)		
	Ever	58	159	0.64 (0.43 to 0.93)		
*GSTP1*						
*AA* (common)	Never	73	143	1.0	0.18	(−0.28 ((−1.10 to 0.62)
	Ever	80	190	0.96 (0.69 to 1.34)		
*AG* or *GG*	Never	75	125	1.29 (0.88 to 1.91)		
	Ever	58	168	1.05 (0.72 to 1.52)		
*GSTA1*						
A*/A* (common)	Never	97	180	1.0	0.41	(−0.17 ((−0.96 to 0.89)
	Ever	82	233	0.67 (0.47 to 0.96)		
A*/B* or B*/B*	Never	54	97	1.07 (0.71 to 1.62)		
	Ever	57	126	0.85 (0.57 to 1.26)		

No. of variants						
Four common	Never	9	24	1.0	0.13	Referent
	Ever	11	44	0.54 (0.26 to 1.15)		
One variant	Never	59	97	1.26 (0.78 to 2.03)		
	Ever	53	122	0.88 (0.55 to 1.42)		0.28 ((−0.39 to 0.45)
Two variants	Never	48	96	1.06 (0.65 to 1.75)		
	Ever	45	126	0.74 (0.45 to 1.21)		(−0.09 ((−0.57 to 0.38)
Three variants	Never	16	27	1.15 (0.56 to 2.35)		
	Ever	15	39	0.79 (0.39 to 1.58)		0.18 ((−0.23 to 0.59)

We adjusted data for age at reference and for season of blood draw. Common alleles: *GSTT1* present, *GSTM1* present, *GSTP1* (*AA*), *GSTA1* (A*/A*). Variant alleles: *GSTT1* null, *GSTM1* null, *GSTP1* (*AG* or *GG*), *GSTA1* (A*/B* or B*/B*).

a From likelihood ratio test.
